# INTERGENERATIONAL BONDING: A PATHWAY TO CAREER READINESS IN SPEECH LANGUAGE PATHOLOGY GRADUATE STUDENTS

**DOI:** 10.1093/geroni/igad104.0473

**Published:** 2023-12-21

**Authors:** Ladan Ghazi-Saidi, Miechelle McKelvey

**Affiliations:** University of Nebraska at Kearney, Kearney, Nebraska, United States; University of Nebraska at Kearney, Kearney, Nebraska, United States

## Abstract

We present a new experiential learning activity for graduate students in two core courses in Speech-Language Pathology. Speech-Language Pathology is a healthcare profession that provides care to people of all ages, including older adults with language, communication, and cognitive disorders. Students typically find these course contents heavy and have limited opportunity to practice the required skills for their future profession. In this multi-faceted program, we provide cognitive intervention to community older adults via telepractice with an intergenerational approach. Students receive real-life interaction to practice the knowledge and skills learned in their academic program. These interactions provide hands-on experience that facilitates better preparation for their future healthcare career. We measure students’ perception of competency pre and post-intervention. Additionally, we survey alumni and their supervisors on students’ preparedness (required skills) and performance during their clinical internship experience. This intergenerational bonding program creates a fun and enjoyable experience that impacts students’ active engagement with older adults. Students report an easier application of academic material, great ownership of learning, and increased motivation to learn new skills. Further, this real-life, interactive experience will impact their career success as future aging healthcare professionals (SLPs).

